# The default mode network as a biomarker for monitoring the therapeutic effects of meditation

**DOI:** 10.3389/fpsyg.2015.00776

**Published:** 2015-06-09

**Authors:** Rozalyn Simon, Maria Engström

**Affiliations:** ^1^Center for Medical Image Science and Visualization, Department of Medical and Health Sciences, Linköping UniversityLinköping, Sweden

**Keywords:** meditation, neuroimaging, default mode network, therapy, mindfulness, functional magnetic resonance imaging (fMRI), biomarker, DMN modulation

## Abstract

The default mode network (DMN) is a group of anatomically separate regions in the brain found to have synchronized patterns of activation in functional magnetic resonance imaging (fMRI). Mentation associated with the DMN includes processes such as mind wandering, autobiographical memory, self-reflective thought, envisioning the future, and considering the perspective of others. Abnormalities in the DMN have been linked to symptom severity in a variety of mental disorders indicating that the DMN could be used as a biomarker for diagnosis. These correlations have also led to the use of DMN modulation as a biomarker for assessing pharmacological treatments. Concurrent research investigating the neural correlates of meditation, have associated DMN modulation with practice. Furthermore, meditative practice is increasingly understood to have a beneficial role in the treatment of mental disorders. Therefore we propose the use of DMN measures as a biomarker for monitoring the therapeutic effects of meditation practices in mental disorders. Recent findings support this perspective, and indicate the utility of DMN monitoring in understanding and developing meditative treatments for these debilitating conditions.

Brain activation, measured by regional blood flow, can be visualized in functional magnetic resonance imaging (fMRI) as the blood-oxygen-level-dependent (BOLD) signal. Since the early years of fMRI research, awake, restful brain activity has been used as a baseline for the measurement of specific tasks. During this baseline brain activation, the synchronous behavior of a number of anatomic regions was observed and initially identified as a network of task-specific deactivations, dubbed the default mode network (DMN; Gusnard and Raichle, 2001; Raichle et al., 2001). Later it was determined that these task specific deactivations of the DMN also showed patterns of coherent activation during periods of rest. These ongoing low-frequency fluctuations in the resting state consume 60–80% of the brain’s energy (Shulman et al., 2004; Raichle and Mintun, 2006) and though observed by many, their significance was not initially understood (Biswal et al., 1995). The DMN became the first, and now the most extensively studied of the many known resting state functional networks. The main nodes of the DMN have been identified as the medial prefrontal cortex (mPFC), anterior and posterior cingulate cortices (ACC, PCC), precuneus (PCU), inferior parietal cortex (IPC), and lateral temporal cortex (Raichle et al., 2001; Raichle and Snyder, 2007). These primary nodes of the DMN are functionally connected, meaning they exhibit concerted fluctuations during functional tasks. The DMN’s robustness has been established both functionally (Shulman et al., 1997; Gusnard and Raichle, 2001; Mazoyer et al., 2001; Raichle et al., 2001; Lazar et al., 2003; Harrison et al., 2008) and structurally (Shulman et al., 2004; Raichle and Mintun, 2006; Greicius et al., 2009). Network activation has been associated with specific mentation including autobiographical memory, self-reflective thought (Gusnard et al., 2001; Sheline et al., 2009), envisioning future events, mind wandering (Mason et al., 2007), and considering the thoughts and perspectives of others (Raichle et al., 2001; Raichle and Snyder, 2007; Buckner et al., 2008).

## The DMN as a Diagnostic Tool

In healthy individuals, DMN activity has been anti-correlated with goal-oriented task-positive networks (TPNs; Fox et al., 2005; Kelly et al., 2008). On the other hand, abnormal DMN activity – such as competitive, antagonistic DMN activation during TPN activity or changes in connectivity between subregions of the DMN – has been associated with a number of psychological disorders such as schizophrenia (Garrity, 2007; Pomarol-Clotet et al., 2008; Camchong et al., 2011; Bastos-Leite et al., 2014), epilepsy (Liao et al., 2010), anxiety (Zhao et al., 2007), depression (Sheline et al., 2009), autism (Assaf et al., 2010), attention deficit hyperactivity disorder (ADHD; Uddin et al., 2008), and Alzheimer’s disease (AD; Greicius et al., 2004; Sheline and Raichle, 2013). These associations have popularized the use of DMN analysis as a method by which to study mental disorders, resulting in a growing body of literature concerning disorder-specific variations within the DMN [see reviews (Greicius, 2008; Broyd et al., 2009; Fox, 2010; Whitfield-Gabrieli and Ford, 2012)]. Some examples of network variation include failure to deactivate the DMN during tasks in both autism (Spencer et al., 2012) and depression (Grimm et al., 2008; Sheline et al., 2009); decreased DMN activity and connectivity in AD (Sorg et al., 2007; Sheline and Raichle, 2013); antagonistic activity during attention-demanding tasks in schizophrenia (Whitfield-Gabrieli et al., 2009); differences in functional connectivity in both anorexia (Cowdrey et al., 2014) and autism (Assaf et al., 2010) and network inhomogeneity in ADHD (Uddin et al., 2008) and bipolar disorders (Liu et al., 2013). In some disorders such as AD (Sperling, 2011; Koch et al., 2012; Balthazar et al., 2014), depression (Li et al., 2013; Wise et al., 2014), and schizophrenia (Shen et al., 2014), these abnormalities of the DMN are consistent enough to be evaluated for use as diagnostic biomarkers.

Amongst these disorders, the relationship between the DMN and AD pathology has been the most thoroughly investigated. Anatomical regions of neuron loss and plaque deposition in AD overlap with regions of the DMN (Buckner et al., 2005). Greicius et al. (2004) observed a decrease in DMN activity and connectivity in patients with AD, likely due to decreased metabolism and physiological disruptions from plaque deposition. According to these analyses, patterns of DMN disruption provide a metric by which to distinguish individual AD subjects from healthy elderly controls with a sensitivity of 85% and a specificity of 77% (Greicius et al., 2004). Work by Sorg et al. (2007) also found patterns of DMN disruption and attenuated activation in patients with mild cognitive impairment (MCI), supporting the use of DMN alterations for the early detection of individuals at risk for AD. Most recently, work by Balthazar et al. (2014) found that by using the PCC as a seed region for DMN functional connectivity analysis, early AD patients could be distinguished from healthy controls with a sensitivity of 77.3 and 70% specificity, indicating that DMN analysis of PCC connectivity could represent a promising biomarker for early AD diagnosis (Sheline et al., 2010b; Sheline and Raichle, 2013).

In contrast to AD which has regions of decreased DMN activity and connectivity, investigations in schizophrenia reveal *increases* in DMN activity during task performance as well as increased connectivity relative to controls (Garrity, 2007; Pomarol-Clotet et al., 2008; Whitfield-Gabrieli et al., 2009; Camchong et al., 2011; Bastos-Leite et al., 2014). In a number of studies, the degree of DMN disorder significantly correlated with the severity of psychological symptoms (Garrity, 2007; Whitfield-Gabrieli et al., 2009; Camchong et al., 2011). The same is true in the case of depression, where abnormal DMN activity and functional connectivity correlate with depressive rumination and symptom severity (Greicius et al., 2007; Berman et al., 2010; Sheline et al., 2010a). Many studies have now established this relationship between DMN-related abnormalities and psychological symptoms such as depressive rumination (Greicius, 2008; Broyd et al., 2009; Sheline et al., 2009; Fox, 2010; Whitfield-Gabrieli and Ford, 2012), feelings of hopelessness (Grimm et al., 2008), mind wandering (Mason et al., 2007), and poor cognitive performance (Weissman et al., 2006; Sorg et al., 2007; Sheline and Raichle, 2013) further intimating the role of the DMN in mental illness.

## The DMN as a Biomarker for Treatment Response

If abnormalities of the DMN can be employed as diagnostic biomarkers or a metric of symptom severity, can the post-treatment normalization of DMN activity and connectivity also be used to evaluate treatment effectivity? Pharmacological fMRI (phfMRI) studies may be the first to provide an answer to this question (Anand et al., 2005; Sambataro et al., 2009; Di Simplicio et al., 2011; Kozel et al., 2011; Tregellas et al., 2011; Li et al., 2012; Andreescu et al., 2013; Posner et al., 2013; Smucny et al., 2014; Wang et al., 2014). For example, Tregellas et al. (2011) used the DMN as a metric in evaluating patient response to medication in schizophrenics with high posterior DMN connectivity and activity. Post-treatment, they found DMN activity resembling healthy network functioning (Tregellas et al., 2011). For the treatment of depression, Wang et al. (2014) reported changes in resting state functional connectivity resulting from the use of antidepressants. Their work also showed that reductions in functional connectivity of the dorsomedial prefrontal cortex, a subregion of the DMN (Sheline et al., 2009), significantly correlated with symptomatic improvement. For the treatment of AD, Li et al. (2012) showed that after administration of Donepezil, patients exhibited increased blood flow and functional connectivity to the PCC region of the DMN, restoring connectivity to levels resembling healthy controls. As a locus for measuring recovery, these changes in the DMN significantly correlated with improved cognitive performance (Li et al., 2012).

The combined use of clinical evaluations with an objective measure such as DMN analysis, could provide a powerful new metric for assessing the success of differing treatments. The essential precursor to this approach is the establishment of reliable, disorder-specific differences between the DMNs of patient populations and healthy controls. In addition, there is evidence supporting the potential to further subgroup broadly diagnosed disorders based on additional DMN variations within patient populations (Lui et al., 2011; Li et al., 2013; Liu et al., 2013). Although these methods are currently being employed to evaluate pharmacological treatments for mental disorders, few have utilized DMN measures to evaluate non-pharmacological cognitive interventions such as meditation. There is a growing body of evidence indicating that meditative mindfulness practices may provide a promising avenue for the treatment of many of the mental disorders discussed above (see reviews Baer, 2006; Chiesa and Serretti, 2009, 2011; Chiesa, 2010; Keng et al., 2011). In this perspective, we propose the use of DMN analysis as an additional objective metric or biomarker for monitoring the therapeutic effects of meditation in mental disorders.

## Meditation and Modulation of the DMN

As research strengthens the link between anatomical regions of the DMN and psychological processes such as self-reflection, rumination, and mind wandering, much interest has been directed toward non-pharmacological means of altering patterns of behavior within this network. Meta-analyses examining the specific neurocorrelates of meditation have shown reductions in DMN activity as a primary outcome of mindfulness meditation practices (Tomasino et al., 2012, 2014). Results from a recent study by Garrison et al. (2015) indicate that meditation is associated with reduced activations in the DMN relative to an active association task for meditators as compared to controls.

For depression, reduced DMN activity in regions associated with subjective evaluation of emotional experience and self-referencing are thought to allow the individual to experience the present moment with greater objectivity, reducing bias or valuation (Ives-Deliperi et al., 2011). A reduction in self-referential evaluation trains the individual to abandon emotionally charged assessments of their internal and external world, thus altering patterns of self-judgment and value assignment (Farb et al., 2007, 2010). Shapiro’s model of mindfulness calls this “re-perceiving” and notes its likeness to psychological models of decentering (Safran, 1990) and detachment (Bohart, 1983). In psychotherapy, dis-identification refers to a process where the individual becomes capable of reappraisal by distinguishing thoughts from feelings. Likewise, through meditation, the patient’s self-perception changes from an enduring entity to a transient entity. In this way the patient becomes less fixated and less likely to ruminate on faults and mistakes (Jackson et al., 2000; Gross, 2002).

Additional psychological benefits of reduced DMN interferences include improvements in attentional control (Lutz et al., 2008; Hasenkamp and Barsalou, 2012; Hasenkamp et al., 2012) by reducing DMN/TPN competition, commonly associated with mental disorders such as schizophrenia (Whitfield-Gabrieli et al., 2009) and ADHD (Sonuga-Barke and Castellanos, 2007; Uddin et al., 2008). These studies associating psychological changes with DMN modulation as an outcome of meditative practice represent only a few of the results motivating recent trends in fMRI research (see **Table 1**).

**Table 1:** Summary of functional magnetic resonance imaging (fMRI) findings on default mode network (DMN) modulation associated with meditation practices.

Meditation	References	DMN findings associated with meditation
Mindfulness	Farb et al. (2007)	Decreased fc between dmPFC and Insula, Decreased mPFC activity during task
FA	Brefczynski-Lewis et al. (2007)	Decreased DMN activations during distraction
LK	Lutz et al. (2008)	Increased activation in mPFC and PCC/precuneus during LK
LK	Engström and Söderfeldt (2010)	Increased activation in mPFC during LK
FA, OM	Manna et al. (2010)	DMN deactivation during FA Precuneus activation during OM
Brain wave Vibration	Jang et al. (2011)	Increased DMN rsfc to mPFC during rest Increased fc between PCC and dACC/dlPFC during rest, FA, OM, and LK
FA, OM, LK	Brewer et al. (2011)	Overall decrease in mPFC and PCC for FA, OM, and LK
FA	Hasenkamp et al. (2012)	Increase in mPFC and PCC during mind wandering
FA	Hasenkamp et al. (2012)	Increased fc between DMN hubs and orbitofrontalcortex/ventromedial PFC during FA
FA	Pagnoni (2012)	Decreased vPMC activity during FA Increased fc vPMC-right temporoparietal junction
FA, OM	Froeliger et al. (2012)	Increased fc DMN-DAN during meditation Increased fc across all networks during rest
Mindfulness	Taylor et al. (2013)	Decreased fc of mPFC to other DMN nodes during rest Increased fc between rIPL and PCC/PCU/dmPFC during rest
FA	Garrison et al. (2013)	PCC deactivations during FA
LK	Garrison et al. (2014)	Decreased PCC/PCU activation during LK Greater fc between PCC/PCU and left inferior frontal gyrus during LK
Acem Non-directive Concentrative	Xu et al. (2014)	Increased DMN activation during Acem non-directive meditation
FA, OM, LK vs. Task	Garrison et al. (2015)	Decreased DMN activity during meditation relative to task

*rs, resting state; fc, functional connectivity; d, dorsal; dm, dorsal medial; dl, dorsal lateral; vPMC, ventral posteromedial cortex; DAN, dorsal attention network; rIPL, right inferior parietal lobe*.

## Mindfulness Meditation as a Clinical Therapy

Westernized forms of meditation stemming from Buddhist traditions have popularized the concept of “mindfulness” as a therapeutic. These Mindfulness methods have been beneficial in the treatment of psychological disorders such as schizophrenia (Chien and Thompson, 2014), depression (Teasdale et al., 2000; Ma and Teasdale, 2004; Eisendrath et al., 2008; Kuyken et al., 2008), addiction (Bowen et al., 2014), alcoholism (Witkiewitz et al., 2005; Garland et al., 2010), anxiety (Grossman et al., 2004; Baer, 2006; Ludwig and Kabat-Zinn, 2008; Shen et al., 2014), MCI (Wells et al., 2013a), and ADHD (Zylowska et al., 2007; Smalley et al., 2009). Preliminary findings suggest the effects of meditation include increases in emotion regulation (Lutz et al., 2014), memory and cognition (Zeidan et al., 2010), self-regulation (Tang et al., 2014), awareness and self-perception (Hölzel et al., 2011b), as well as gray and white matter differences in experienced meditators (Luders et al., 2009; Hölzel et al., 2011a; Tang et al., 2012, 2015; Fox et al., 2014). Mindfulness-based stress reduction (MBSR) programs incorporate meditation techniques with group meetings, simple yoga, and home assignments (Teasdale et al., 2000; Kabat-Zinn, 2003). This approach allows for broad applications as these programs are standardized and have no religious associations, making them suitable for researchers and clinicians. Though the religious associations of traditional forms of meditative practice have been reduced through such westernized approaches, the number of variables in Mindfulness programs still confound evaluation of clinical efficacy (Chiesa and Serretti, 2009, 2011; Chiesa, 2010). Understanding the specific mechanisms of mindfulness that lead to multi-dimensional mental health outcomes is no trivial task. The reduction of mindfulness into measurable components has been suggested by many resulting in a number of different psychometric assessments (Brown, 2004; Grossman et al., 2004; Feldman et al., 2006; Lau et al., 2006; Baer et al., 2008). In addition to the identification of specific outcomes, meditation efficacy studies have also proven challenging due to variations in meditative practice, length of time practiced, lack of controls, and the inability to conduct double-blind studies – a fault inherent to all therapy efficacy studies. Recent studies using active controls for MBSR programs (MacCoon et al., 2012, 2014; Rosenkranz et al., 2013) have improved upon previous investigations which only used wait list control groups. These types of experimental improvements are necessary to distinguish the specific effects resulting from meditation training versus group therapy or other forms of general behavioral modification. These studies highlight the need for more controlled experimental design and specific biomarkers such as the DMN by which to follow the underlying neurological changes associated with meditation training.

As with all emerging fields, consensus building to bridge areas of specialization requires time and a sufficient amount of preliminary data. These issues are widely recognized, leading to the development of a more defined theoretical framework (Hölzel et al., 2011b) and improved operational definitions (Bishop et al., 2004; Shapiro et al., 2006). These improvements help to guide fMRI experiment design in order to provide objective, empirical evaluation of this previously elusive, highly internalized process.

Despite these improvements, variations in meditative methods persist as a barrier in the advancement of this line of research. A majority of the techniques used in experimental approaches are based in Buddhist traditions. Three of the fundamental practices central to nearly all Buddhist meditation have been generalized to include other traditions, and redefined by some researchers as focused attention (FA), open monitoring (OM), and loving kindness (LK), respectively (Lutz et al., 2004, 2008). These are well-developed techniques commonly encountered in research literature which will only briefly be described here, but whose histories, grounding philosophies, and complete descriptions are reviewed extensively by Lutz, Dunne, and Davidson (Lutz et al., 2007). While it is essential for general research purposes that FA, OM, and LK be investigated separately in terms of their DNM-associated neurocorrelates, we hypothesize that the synergy of all three practices is key to cultivating “equanimity” and is essential when considering meditation as a form of general therapy for mental disorders (Desbordes et al., 2014).

During FA, the participant sits calmly with all attention focused on some object of interest, commonly the breath. Each time the mind begins to wander, the meditator is trained to guide the focus of attention back with non-judgmental awareness. This practice is meant to develop the individual’s meta-awareness, focus, and attention – skills required for all subsequent meditative practices. FA practice can be viewed as mental training to reduce the competitive distraction and daydreaming activities of the DMN. Hasenkamp et al. (2012) investigated the neurocorrelates of fluctuating FA phases in experienced meditators. Participants were asked to maintain FA on breath and instructed to press a button when they realized their attention had wandered. Activity could be detected in brain regions associated with FA, mind wandering, and awareness of mind wandering. DMN activations were correlated with periods of mind wandering, contrasting with attentional activations during awareness, shifting, and maintaining FA. A follow-up resting state study revealed that connectivity in attentional networks correlated with hours of meditative experience, indicating that the repeated process of refocusing the attention leads to increases in attentional control and reduced distractibility of the practitioner in everyday life (Hasenkamp and Barsalou, 2012). This study thus supports the use of FA as a form of training to reduce DMN/TPN competition. Work by Brefczynski-Lewis et al. (2007) supports this hypothesis of decreased distractibility with increased meditative practice. They found that while listening to distractive sounds, relative to novices, expert meditators had less activation in regions within the DMN and more activation in regions related to response inhibition and attention. These results support the hypothesis that the mental health benefits of FA meditation are a result of the cultivation of attentional control through trained disengagement of the DMN, reducing related mentation associated with rumination (Berman et al., 2010; Sheline et al., 2010a), mind wandering (Mason et al., 2007), and unhappiness (Killingsworth and Gilbert, 2010). The practice of FA lays a foundation for all subsequent techniques, as distractibility is detrimental to meditative practice.

The second common technique of mindfulness practice is OM, where the meditator directs their attention toward the non-judgmental awareness of internal and external physical sensations. The meditator’s attention is expected to wander, yet the individual neither cultivates nor forcefully suppresses distracting thoughts. In OM, the meditator is instructed to non-judgmentally observe thoughts and sensations while remaining unreactive. This trains the individual to reduce emotional reactivity and volatility. The objective in OM is to develop insight into the subjective and constantly changing nature of reality while maintaining present awareness. For beginners, establishing oneself in OM meditation often begins with some form of FA. However, this requirement is thought to depend on hours of experience (Brewer et al., 2011). Manna et al. (2010) observed an increase in activation of the PCU, a hub of the DMN, during OM when compared to FA. This study, which investigated differences between OM and FA meditation, found that when compared to novices, expert practitioners’ patterns of brain activity during OM resembled their normal resting state activity. They postulated that with extended practice, this state of non-judgmental awareness or “mindfulness” becomes the intrinsic or default mode of brain activity (Manna et al., 2010).

Although some amount of mind wandering during OM is accepted, identifying with, attaching to, or engaging in these thoughts is discouraged. We suggest that in this way, one develops an ability to be simultaneously present and aware of physical stimuli while engaging in DMN-associated creative processes such as reflection and mind wandering without emotional attachment or reactivity. Put another way, training to suppress the DMN through FA allows for the gradual and controlled reintroduction of DMN-related activities in OM with an enhanced metacognition, abrogating uncontrolled emotional reactivity and self-identification.

The third common element of most Buddhist meditation practices is LK, or LK, where the meditator focuses on feelings of LK and compassion toward others. During LK meditation, all forms of stimuli can be called upon, such as visualization, memory, self-reflection, and auditory mantras, in an effort to dissolve feelings of separation, isolation, and conflict between the meditator and others. This process utilizes skills developed in both FA and OM meditation. This element of Buddhist practice was not included in the proposed operational definition of mindfulness by Bishop et al. (2004). For treatment of psychological disorders however, there is a strong argument for the inclusion of this practice as the objective is to develop empathy and compassion for oneself and others (Hofmann et al., 2011; Farb et al., 2012; Shonin et al., 2014). A study by Garrison et al. (2014) showed that DMN connectivity was reduced during LK meditation, possibly as a result of reduced self-referential processing. Related findings investigating the neurocorrelates of empathy and forgiveness have observed DMN activations in the PCC/PCU (Farrow et al., 2001; Völlm et al., 2006). Engström and Söderfeldt (2010) found activations in the mPFC during LK meditation which others propose reflects processing which is important for experiencing empathy (Seitz et al., 2006). The basic link between the practice of empathy and the mental processes associated with the DMN – considering the perspective of others, autobiographical memory, and self-referential thought – seems self-evident. However, much work remains to define the specific changes in the DMN resulting from this form of meditative training.

Across all three types of meditation, Brewer et al. (2011) observed that two primary nodes of the DMN, the PCC and mPFC, were less active in experienced meditators compared to novices. Thus, for experienced meditators relative to novices, DMN processes such as mind wandering are reduced even before task engagement. In addition, they found increased functional connectivity in experienced meditators between the PCC and task-positive regions during all conditions including rest, indicating trait-based neural differences in long-term meditators. Like Manna et al. (2010) they suggest that long-term meditative practice may transform the individual’s intrinsic resting state into a more “present-centered” meditative state (Brewer et al., 2011).

## First Steps: Clinical Trials

**FIGURE 1:**
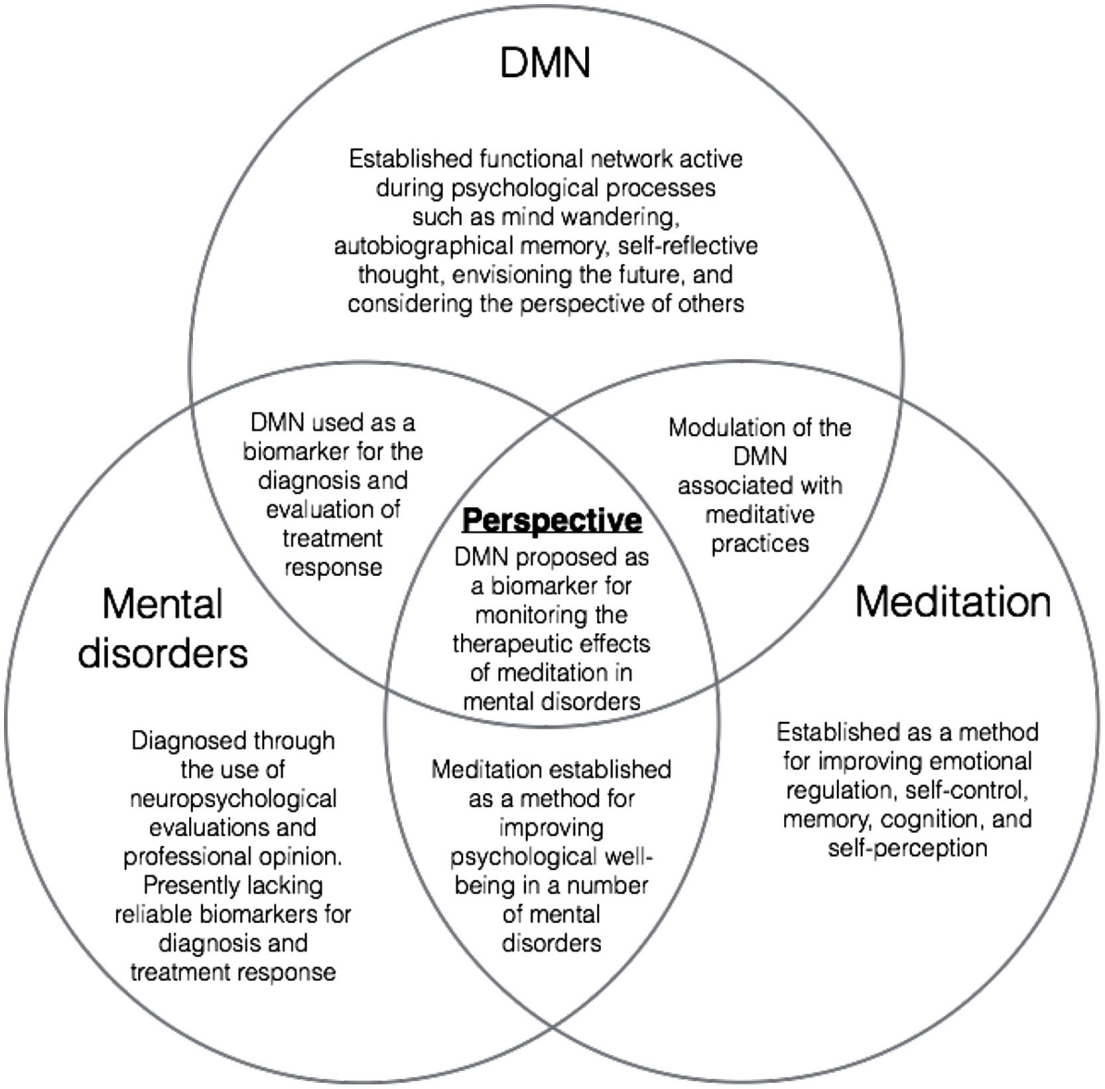
**A visual representation of overlapping areas of research converging on the present perspective**.

Taken together, these studies provide evidence to support further investigation into the use of DMN metrics for the evaluation of meditative therapies (see **Figure 1**). Indeed, controlled studies correlating resting state DMN modulation with neuropsychological measures for the evaluation of Mindfulness therapies are emerging with promising results. Wells et al. (2013b) conducted a pilot study to investigate the effects of MBSR training on a group of mild cognitively impaired (MCI) patients at risk for AD. The results indicated that after MBSR training, MCI patients had increased DMN connectivity in the PCC, mPFC, and hippocampus, relative to controls. They also investigated changes in the volume of the hippocampus, a region known to atrophy in MCI/AD, and found trends toward less hippocampal atrophy in MBSR-trained patients relative to controls. As a result of these findings they suggest that DMN connectivity could be used as a non-invasive biomarker for assessing the impact of mindfulness interventions in MCI patients, though larger studies need to be conducted (Wells et al., 2013b).

Ives-Deliperi et al. (2013) conducted a controlled study investigating the effects of Mindfulness-based Cognitive Therapy (MBCT) in bipolar disorder. Previous to treatment, bipolar patients had significantly higher levels of stress and anxiety, lower scores on working memory tasks, and decreases in mPFC signals during task relative to healthy controls. After treatment, they observed BOLD increases in the mPFC and posterior parietal lobe during task, significant increases in mindfulness measures and working memory, as well as decreases in anxiety and emotional dysregulation. Region of interest analysis verified a correlation between mindfulness measures and mPFC increases in BOLD signals. This study provides additional support that the DMN represents a suitable network by which to assess the effects of meditative therapies in the treatment of mental disorders (Ives-Deliperi et al., 2013).

## Future Perspectives

These recent findings demonstrate use of the DMN as a potentially useful clinical tool for evaluating the therapeutic effects of meditation. Research to establish disorder-specific DMN irregularities is a necessary first step. Determining DMN differences relative to healthy controls allows for an objective measure of patient recovery or return to control-like activity and connectivity within the DMN. Correlation with neuropsychological tools for measuring symptomatic changes and cognitive improvement will help to guide the development of this emerging tool and to define its significance. However, many experimental design issues still confound the evaluation of clinical efficacy (Tang et al., 2015). First, reducing the number of coinciding therapies in Mindfulness programs and having proper control groups is essential. Documenting explicit instructions for the meditation practice will also be important for comparative studies and meta-analysis. Future studies could also include analysis of the dynamic functional connectivity associated with mental disorders and treatment response. This may allow for further subgrouping under broad diagnoses and predictions concerning treatment outcomes. Although the DMN is the best studied of the resting state networks, the analysis of other networks should also be investigated (Froeliger et al., 2012). In addition, the development and incorporation of new neuropsychological metrics – such as “equanimity” outcomes – with resting state correlates will provide new tools for assessment (Desbordes et al., 2014). Finally, finding ways to integrate objective data and subjective patient reporting will be useful in understanding patient experiences associated with meditative practices (Garrison et al., 2013; Brewer and Garrison, 2014; Hasenkamp, 2014). It seems fitting that a method as internalized as meditation would be useful in treating conditions associated with neural processes so deeply intrinsic as to be called the “default mode” of brain function.

## Conflict of Interest Statement

The authors declare that the research was conducted in the absence of any commercial or financial relationships that could be construed as a potential conflict of interest.
